# Supplementary material for: Quantum neural network cost function concentration dependency on the parametrization expressivity
Source: Sci Rep. 2023 Jun 20;13:9978. doi: 10.1038/s41598-023-37003-5 (PMC10281952; doi:10.1038/s41598-023-37003-5)
Supplement: Supplementary file 1 — Supplementary Information. [file 41598_2023_37003_MOESM1_ESM.pdf]

Supplementary information for "Quantum neural network cost function concentration dependency on the parametrization expressivity"

Lucas Friedrich, and Jonas Maziero

Physics Departament, Center for Natural and Exact Sciences,
Federal University of Santa Maria, Roraima Avenue 1000, 97105-900, Santa Maria, RS, Brazil

In this supplementary information, we provide detailed proofs of theorems 1 and 2. Furthermore, we present the different parameterization models used in this work.

VII. PROOF OF THEOREM 1

For the proofs we present in this and in the next section, we shall need the following lemmas:

Lemma 1 *Let $\{W_y\}_{y\in Y}\subset U(d)$ form a unitary t-design with $t\geq 1$, and let $A,B:H_w\rightarrow H_w$ be arbitrary linear operators. Then*

$$\int d\mu(W)Tr[WAW^{\dagger}B]=\frac{Tr[A]Tr[B]}{d}. \tag{S1}$$

Lemma 2 *Let $\{W_y\}_{y\in Y}\subset U(d)$ form a unitary t-design with $t\geq 2$, and let $A,B,C,D:H_w\rightarrow H_w$ be arbitrary linear operators. Then*

$$\begin{aligned}\int d\mu(W)Tr[WAW^{\dagger}B]Tr[WCW^{\dagger}D]=\\ =\frac{Tr[A]Tr[B]Tr[C]Tr[D]+Tr[AC]Tr[BD]}{d^2-1}\\ -\frac{Tr[AC]Tr[B]Tr[D]+Tr[A]Tr[C]Tr[BD]}{d(d^2-1)},\end{aligned} \tag{S2}$$

where $d=2^n$. For more details on the proofs of these lemmas, see Refs. [1, 2].

We will also use the definition of expressivity given in Eq. (3). For proving Theorem 1, we start by writing the mean of the cost function as in Eq. (6). Using $A(\rho):=A^1_{\mathbb{U}}(\rho)$ given by Eq. (3) with $t=1$ in Eq. (6), we get

$$E_{\mathbb{U}}[C]=\int d\mu(V)Tr[OV\rho V^{\dagger}]-Tr[OA(\rho)]. \tag{S3}$$

Using the cyclicity of the trace function and Lemma 1, we obtain

$$E_{\mathbb{U}}[C]=\frac{Tr[O]}{d}-Tr[OA(\rho)], \tag{S4}$$

where we used that $Tr[\rho]=1$. From Eq. (S3), it follows that

$$\begin{aligned}\left|E_{\mathbb{U}}[C]-\frac{Tr[O]}{d}\right|&=|Tr[OA(\rho)]|\\ &\leqslant \|O\|_2\|A(\rho)\|_2.\end{aligned} \tag{S5}$$

Above we used the Cauchy-Schwarz inequality. With this, we have proved Theorem 1.

VIII. PROOF OF THEOREM 2

To prove Theorem 2, we start by writing the variance as

$$Var_{\mathbb{U}}[C]=E_{\mathbb{U}}[C^2]-E_{\mathbb{U}}[C]^2, \tag{S6}$$

where $E_{\mathbb{U}}[C]$ has already been obtained in Eq. (S3). Therefore, we get $E_{\mathbb{U}}[C^2]$, which will be given by

$$\begin{aligned} E_{\mathbb{U}}[C^2] &= \int dU (Tr[OU\rho U^\dagger])^2 \\ &= \int dU Tr[O^{\otimes 2} U^{\otimes 2} \rho^{\otimes 2} (U^\dagger)^{\otimes 2}]. \end{aligned} \tag{S7}$$

Thus, using $A^{\otimes 2}(\rho^{\otimes 2})$ in Eq. (S7), with $A^{\otimes 2} = A^2_{\mathbb{U}}$, we get

$$\begin{aligned} E_{\mathbb{U}}[C^2] = \int d\mu(V) Tr[O^{\otimes 2} V^{\otimes 2} \rho^{\otimes 2} (V^\dagger)^{\otimes 2}] \\ - Tr[O^{\otimes 2} A^{\otimes 2}(\rho^{\otimes 2})]. \end{aligned} \tag{S8}$$

To solve the integral that appears in Eq. (S8), we use Lemma 2. So

$$\begin{aligned} &\int d\mu(V) Tr[OV\rho V^\dagger] Tr[OV\rho V^\dagger] \\ &= \frac{Tr[O]^2 + Tr[O^2]}{d^2 - 1} - \frac{Tr[O]^2 + Tr[O^2]}{d(d^2-1)} \\ &= \frac{Tr[O]^2 + Tr[O^2]}{d^2 - 1}\left(1 - \frac{1}{d}\right), \end{aligned} \tag{S9}$$

where we again used the cyclicity of the trace function. We also used $Tr[\rho] = 1$ and $Tr[\rho^2] = 1$.
Using this result in Eq. (S8), we get

$$E_{\mathbb{U}}[C^2] = \frac{Tr[O]^2 + Tr[O^2]}{d^2 - 1}\left(1 - \frac{1}{d}\right) - Tr[O^{\otimes 2} A^{\otimes 2}(\rho^{\otimes 2})]. \tag{S10}$$

Using the results obtained in Eq. (S5) and (S10), we have that the variance will be given by

$$\begin{aligned} Var_{\mathbb{U}}[C] = \frac{Tr[O]^2 + Tr[O^2]}{d^2 - 1}\left(1 - \frac{1}{d}\right) - Tr[O^{\otimes 2} A^{\otimes 2}(\rho^{\otimes 2})] \\ - \left[\frac{Tr[O]}{d} - Tr[OA(\rho)]\right]^2 \end{aligned} \tag{S11}$$

or

$$\begin{aligned} Var_{\mathbb{U}}[C] = \beta - Tr[O^{\otimes 2} A^{\otimes 2}(\rho^{\otimes 2})] \\ + \alpha Tr[OA(\rho)] - Tr[OA(\rho)]^2 \end{aligned} \tag{S12}$$

with

$$\beta := \frac{Tr[O]^2 + Tr[O^2]}{d^2 - 1}\left(1 - \frac{1}{d}\right) \tag{S13}$$

and

$$\alpha := \frac{2Tr[O]}{d}. \tag{S14}$$

Therefore, from Eq. (S12), we get

$$\begin{aligned} |Var_{\mathbb{U}}[C]| &\leqslant |\beta| + |Tr[O^{\otimes 2} A^{\otimes 2}(\rho^{\otimes 2})]| \\ &\quad + |\alpha Tr[OA(\rho)]| + |Tr[OA(\rho)]^2| \\ &\leqslant |\beta| + \|O^{\otimes 2}\|_2 \|A^{\otimes 2}(\rho^{\otimes 2})\|_2 \\ &\quad + |\alpha| \|O\|_2 \|A(\rho)\|_2 + \|O\|_2^2 \|A(\rho)\|_2^2, \end{aligned} \tag{S15}$$

where the first inequality was obtained from the triangular inequality for complex numbers and the second inequality is obtained from the Cauchy-Schwarz inequality. In addition, we use $|x^2| = |x|^2$ for $x \in \mathbb{R}$. With this, we complete the proof of Theorem 2.

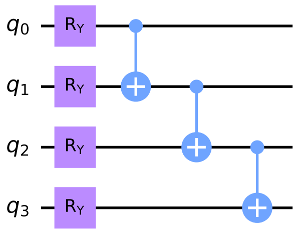

Figure S1: Model 1.

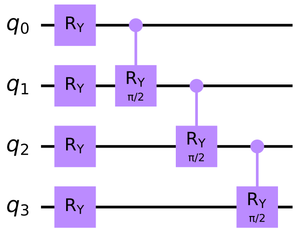

Figure S2: Model 2.

IX. QUANTUM CIRCUITS FOR THE SIMULATIONS

For all simulations that we presented in main manuscript, we used the parametrizations shown in Figs. S1, S2, S3, S4, S5, S6, S7, S8, S9, S10, S11, and S12.

References

[1] B. Collins and P. Śniady, Integration with respect to the Haar measure on unitary, orthogonal and symplectic group, Commun. Math. Phys. 264, 773 (2006).

[2] Z. Puchała and J. A. Miszczak, Symbolic integration with respect to the Haar measure on the unitary group, Bull. Pol. Acad. Sci.-Tech. Sci. 65, 1 (2017).

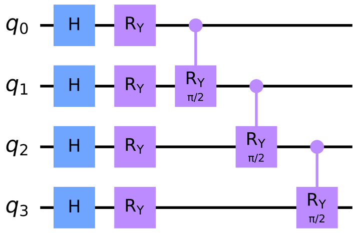

Figure S3: Model 3.

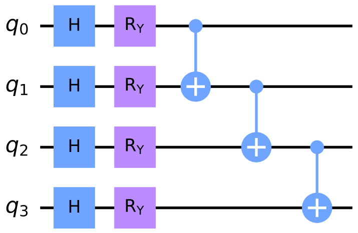

Figure S4: Model 4.

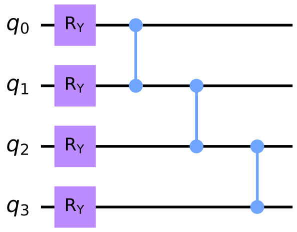

Figure S5: Model 5.

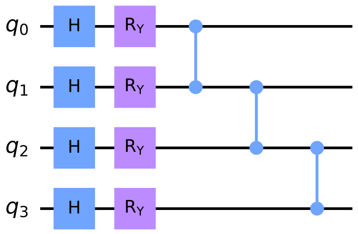

Figure S6: Model 6.

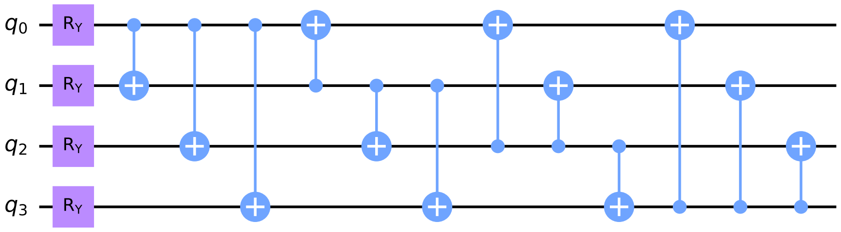

Figure S7: Model 7.

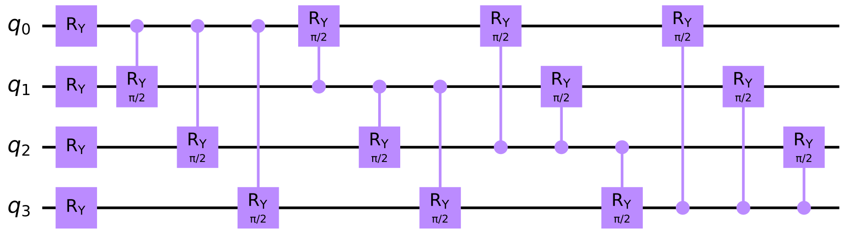

Figure S8: Model 8.

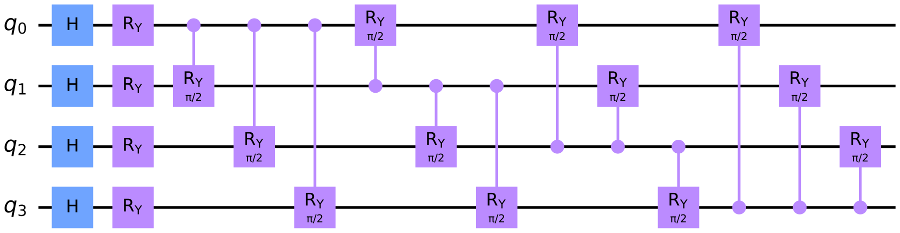

Figure S9: Model 9.

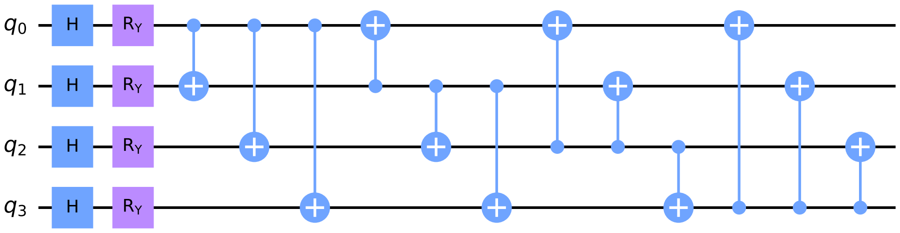

Figure S10: Model 10.

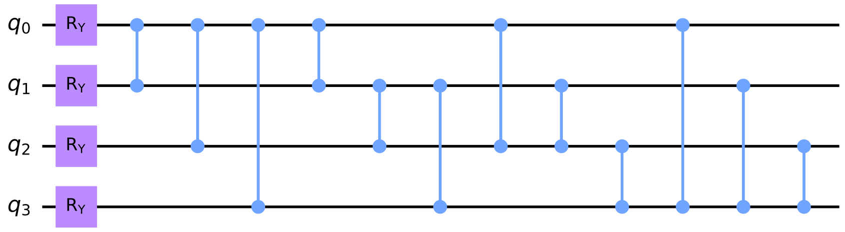

Figure S11: Model 11.

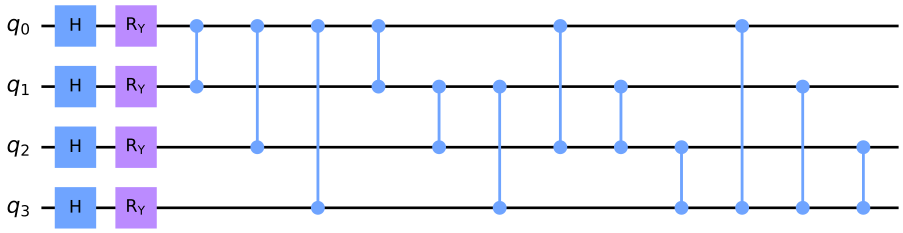

Figure S12: Model 12.
